# Localization studies of two white spot syndrome virus structural proteins VP51 and VP76

**DOI:** 10.1186/1743-422X-3-76

**Published:** 2006-09-12

**Authors:** Chenglin Wu, Feng Yang

**Affiliations:** 1 Key Laboratory of Marine Biogenetic Resources, Third Institute of Oceanography, 178 Daxue Road, Xiamen, P.R. China

## Abstract

VP51 and VP76 are two structural proteins of white spot syndrome virus (WSSV). However, there is some controversy about their localization in the virion at present. In this study, we employ multiple approaches to reevaluate the location of VP51 and VP76. Firstly, we found VP51 and VP76 presence in viral nucleocapsids fraction by Western blotting. Secondly, after the high-salt treatment of nucleocapsids, VP51 and VP76 were still exclusively present in viral capsids by Western blotting and immunoelectron microscopy, suggesting two proteins are structural components of the viral capsid. To gather more evidence, we developed a method based on immunofluorescence flow cytometry. The results revealed that the mean fluorescence intensity of the viral capsids group was significantly higher than that of intact virions group after incubation with anti-VP51 or anti-VP76 serum and fluorescein isothiocyanate conjugated secondary antibody. All these results indicate that VP51 and VP76 are both capsid proteins of WSSV.

## Background

White spot syndrome virus (WSSV), the only species of the genus *Whispovirus *of the family *Nimaviridae*, is one of most virulent viral disease known in the shrimp farming industry around the world, which also infect most species of crustacean, such as crabs and crayfish [1-3]. Studies have shown that WSSV virion is an ellipsoid shape enveloped particle and it has a bacilliform nucleocapsid which is similar to insert baculovirus. The most obvious feature of WSSV is the presence of a long, tail-like extension at one end of the virion [4-9].

Up to now, the complete genome sequences of three isolates (WSSV-CN, WSSV-TH and WSSV-TW) have been sequenced [10-12] and many structural proteins of WSSV have been identified by combining SDS-PAGE with mass spectrometry (MS) or two-dimensional electrophoresis with MS [13-15], some of which have been confirmed to be envelope proteins by Western blotting and immunoelectron microscopy (IEM) including: VP24 [16], VP26/P22 [16,17], VP28 [18], VP31 [19], VP36/VP281 [20], VP39 [21], VP124 [22], VP187 [23] and VP110 [24]. However, the nucleocapsid proteins of WSSV are less well understood, except VP15 and VP664. VP15 is a basic DNA binding protein located in WSSV nucleocapsid and similar to histone [25,26]. VP664, the largest protein of WSSV, containing 6077 aa, was reported to encode a major nucleocapsid protein VP664 [27].

Recently, we noticed that two WSSV structural proteins, VP51 and VP76, seemed to be present in viral nucleocapsid fraction [28]. In addition, VP51 was also thought to be viral nucleocapsid proteins by immunoblotting in recent study [29]. However, in previous studies, VP51 and VP76 were reported as viral envelope proteins. VP51 was considered as a viral envelope protein by IEM after 18 structural proteins from the virions using MS were identified [13]. Likewise, VP76 was believed as viral envelope protein by Western blotting [30]. Because the localization of the two proteins is controversial at present, a more precise identification is necessary to perform functional studies in future. In this investigation, we employ multiple approaches to clarify the location of VP51 and VP76, and all experimental evidence indicated that VP51 and VP76 are viral capsid proteins.

## Results and discussion

### Identification of VP51 and VP76 by Western blotting

**Figure 1:**
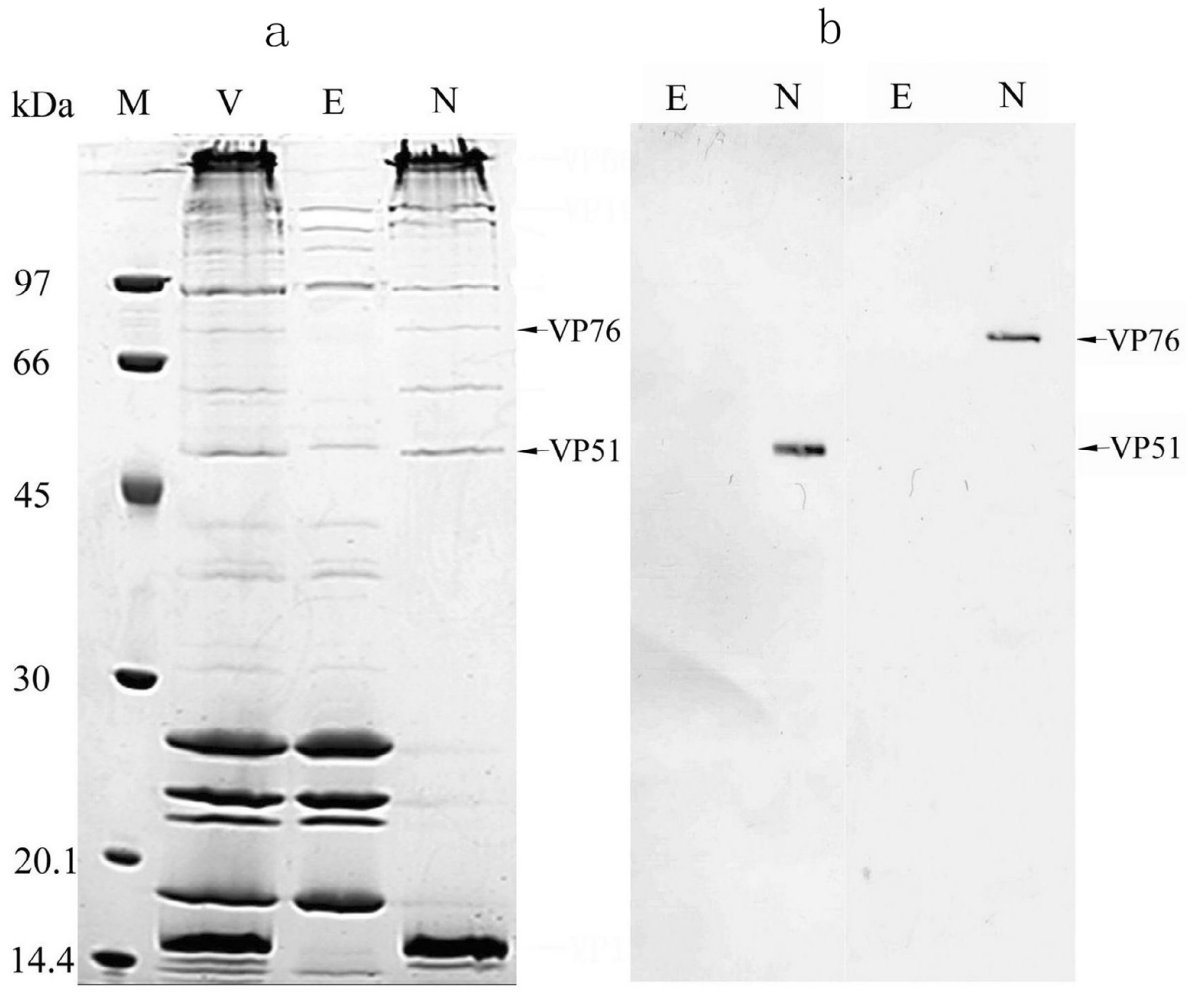
a. Coomassie brilliant blue-stained 12% SDS-PAGE of the various fractions of WSSV. Lanes: M, low molecular mass protein marker; V, intact virsions; E, envelope fraction after Triton X-100 treatment; N, nucleocapsid fraction after Triton X-100 treatment. VP51 and VP76 are indicated by arrows. b. Western blot analysis with anti-VP51 or VP76 serum respectively. Lanes: E, envelope fraction after Triton X-100 treatment; N, nucleocapsid fraction after Triton X-100 treatment. VP51 and VP76 are indicated by arrows.

The truncated recombinant proteins, VP51p and VP76p, were expressed in *Escherichia coli *and purified by using Ni-NTA affinity chromatography, and then specific antiserum against VP51 and VP76 were obtained by immunizing mice. To perform localization studies, WSSV virions, envelope and nucleocapsid fractions were separated by SDS-PAGE, followed by staining with Coomassie brilliant blue R-250. As shown in Fig. 1a, at least 8 distinct protein bands were revealed from the viral nucleocapsid fraction, including two of them with apparent molecular mass of about 51 and 76 kDa. Whilst, Western blotting analysis indicated that the two proteins were exclusively detected in nucleocapsid fraction (Fig. 1b).

**Figure 2:**
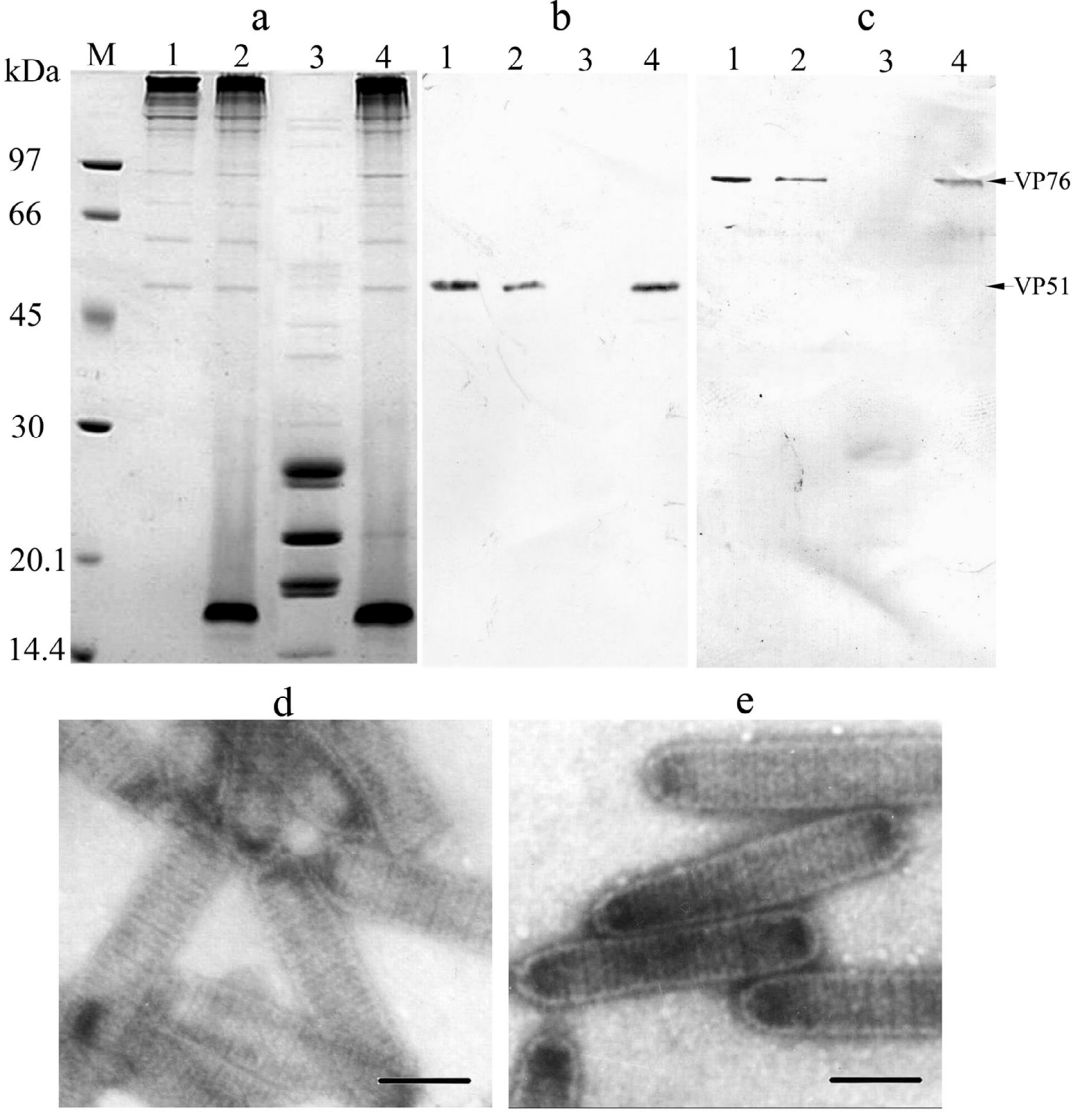
a. Coomassie brilliant blue-stained 12% SDS-PAGE of the nucleocapsid and envelope proteins of WSSV. Lanes: M, low molecular mass protein marker; 1, high-salt treated nucleocapsid sample; 2, low-pH treated nucleocapsid sample. b, c. Western blot analysis with anti-VP51 or VP76 serum respectively. 1, high-salt treated nucleocapsid sample; 2, low-pH treated nucleocapsid sample; 3, envelope fraction after Triton X-100 treatment; 4, nucleocapsid fraction after Triton X-100 treatment. d, e. TEM examination of high-salt or low-pH treated nucleocapsid sample, respectively. Bars, 100 nm.

To eliminate the possible interference of the remaining viral envelope proteins, Triton-treated nucleocapsids were further treated under more stringent conditions (high-salt or low-pH). The results of SDS-PAGE analysis shown the protein bands of 51 and 76 kDa were still observed clearly after high-salt or low-pH treatment (Fig. 2a), whilst Western blot analysis also revealed the same results using anti-VP51 or VP76 serum (Fig. 2b, c, line1, 2 and 4). Above results suggest that VP51 and VP76 are associated with virus nucleocapsids.

In addition, during high-salt treatment, we observed that the nucleocapsid suspension became very thick in comparison with the result of low-pH treatment, and VP15 was completely removed from viral nucleocapsid fraction after the treatment (Fig. 2a, lane 1), but TEM results showed that high-salt treated sample still retained its integrality in configuration (Fig. 2d). Therefore, we conclude that high-salt treatment can lead to release of viral genomic DNA fibres and VP15 from viral nucleocapsid particles, and so the image of particles observed under electron microscope actually is viral capsids. The above experiments indicated that VP51 and VP76 are likely the viral minor capsid proteins, suggesting that WSSV capsid particles can be purified by high-salt treatment of nucleocapsids.

### Localization of VP51 and VP76 by IEM

**Figure 3:**
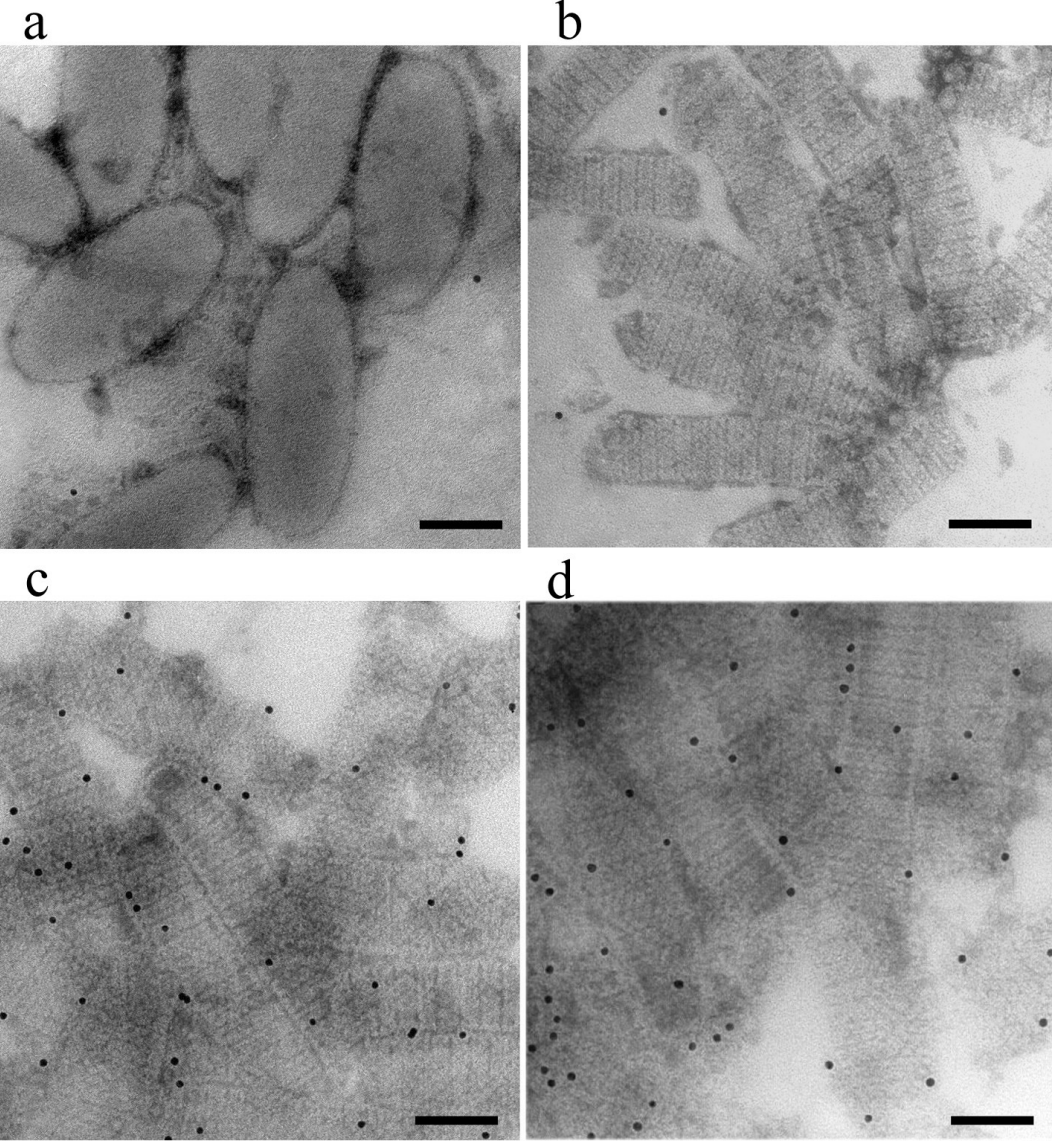
Localization of VP51 and VP76 in WSSV by IEM. (a) Intact virions with anti-VP51 or anti-VP76 serum; (b) high-salt treated nucleocapsid sample with non-immune mouse serum; (c) high-salt treated nucleocapsid sample with anti-VP51 serum; (d) high-salt treated nucleocapsid sample with anti-VP76 serum. Bars, 100 nm.

To verify above results, we performed immunoelectron microscopy localization studies by means of an indirect immunogold labelling method using anti-VP51 or VP76 antibodies. The results showed that no gold particles could be seen on purified WSSV virions when using anti-VP51 or anti-VP76 serum as the primary antibody (Fig. 3a), whereas under same condition, gold particles were markedly distributed on high-salt treated nucleocapsids, viz. capsids (Fig. 3c, d). Control experiments showed that no gold particles were found on the capsids when using non-immune mouse serum as the primary antibody (Fig. 3b). The IEM results indicated that VP51 and VP76 were located on the viral capsid, suggesting that they are structural components of the viral capsid.

### Localization of VP51 and VP76 by FCM

**Figure 4:**
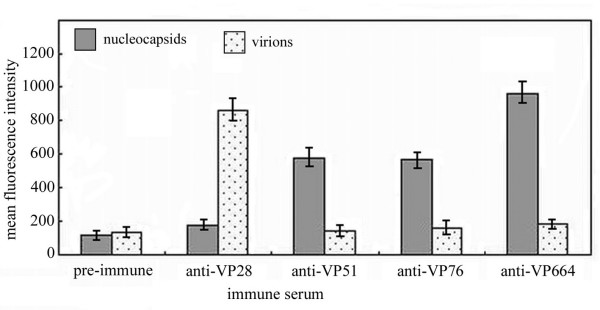
Comparison of mean fluorescence intensity between WSSV capsid group (high-salt treated) and virion group stained with different antiserum (in order: non-immune mouse, anti-VP28, anti-VP51, anti-VP76 and anti-VP664 serum) by immunofluorescence flow cytometry. Data are expressed as the means ± standard deviation of three independent experiments.

It is well known that FCM could be used to analyze cell surface marker by means of a fluorescent labeled specific antibody. In order to gather more evidence to make a credible conclusion, we developed a practical method based on immunofluorescence flow cytometry to study the localization of VP51 and VP76 by comparing the mean fluorescence intensity of the population of intact virions or high-salt treated nucleocapsids (capsids). This notion derives from reports that virus particles could be counted directly by FCM, such as the quantification of marine viruses [31,32] and the detection of baculovirus [33-35]. Although WSSV is small relatively in size in comparison with cells, their size (virion: ~ 275 × 120 nm; nucleocapsid: ~ 300 × 70 nm, [9]) is enough to be detected by FCM. In this experiment, WSSV virion and capsid groups were incubated with anti-VP51 or anti-VP76 serum, followed by staining with FITC-conjugated goat anti-mouse IgG. The analysis results showed that the fluorescent signal of the viral capsid group was significantly higher than the virion group (Fig. 4). To ensure reliability of data, anti-VP664 or anti-VP28 serum was conducted as primary antibody in positive control experiments. As expected, a stronger fluorescent signal was observed in the capsid group of anti-VP664 serum treatment, whereas the fluorescence intensity from virion group was low, which only corresponded to the background signal caused by pre-immune serum. In contrast, the virion group displayed much strong fluorescence intensity by using anti-VP28 serum. This data further support the conclusion from Western blotting or IEM. Based on the experiment, we considered FCM is an effective alternative technique for the localization of the structural proteins of WSSV or other large viruses due to its facility, high efficiency and sensitivity.

All in all, WSSV is most virulent pathogen of the penaeid shrimp farming industry. But until now, the pathogenesis of WSSV has not been clearly understood on the molecular level. Thus there is an urgent need to study the structural proteins and their function of this virus to find out the solution to prevent or cure this disease. In this paper, we performed localization studies of two viral structural proteins, VP51 and VP76, in WSSV virions by employing multiple approaches, and make a definite conclusion, i.e. VP51 and VP76 reside in the viral nucleocapsid, and are viral minor capsid proteins. The results may facilitate a better understanding of the molecular mechanism of WSSV infection and assembly, or be helpful for the control of virus infection in the future.

## Conclusion

The localization of the two proteins, VP51 and VP76, is controversial at present. In this investigation, we employ multiple approaches (Western blotting and IEM, as well as flow cytometry etc.) to clarify the location of VP51 and VP76, and all experimental evidence indicated that VP51 and VP76 reside in the viral nucleocapsid, and are viral minor capsid proteins. We considered FCM is an effective alternative technique for the localization of the structural proteins of WSSV or other large viruses due to its facility, efficiency and sensitivity.

## Materials and methods

### Preparation of intact WSSV virions and nucleocapsids

WSSV virions were prepared essentially as described previously [28]. Briefly, WSSV-infected crayfish tissues were homogenized, and then centrifuged at 3500 × *g *for 5 min at 4°C. After filtering by nylon net (400 mesh), the supernatant was centrifuged at 30,000 × *g *for 30 min at 4°C. Then, the upper loose layer (pink) of pellet was rinsed out carefully using a Pasteur pipette, and the lower compact layer (gray) was resuspended in TM buffer (50 mM Tris-HCl/pH7.5, 10 mM MgCl_2_). After several rounds of conventional differential centrifugations, the milk-like pure virus suspension was obtained and stored at 4°C until use.

Separation of envelope and nucleocapsid fractions was carried out as described recently with slight modifications [16]. In brief, a 0.5 ml pure virus suspension was mixed with an equal volume of 2% Triton X-100 and then incubated for 30 min at room temperature with gentle shaking. The nucleocapsids were purified by centrifugation at 20,000 × *g *for 20 min at 4°C. The envelope proteins (the supernatant) were collected and used for the following experiments, whilst the pellet (nucleocapsids) was subjected to a second round of Triton X-100 extraction to ensure complete treatment. Finally, the Triton-treated nucleocapsids were suspended in 1 ml of TM buffer and stored at 4°C until use.

### Retreatment of nucleocapsids by high-salt or low-pH buffer

High-salt treatment: in general, a 0.2 ml Triton-treated nucleocapsid suspension was mixed with 0.8 ml of TNK buffer (20 mM Tris-HCl/pH7.6, 0.8 M NaCl, 0.8 M KCl), and mixture incubated for 30 min at 4°C. The mucous mixture was centrifuged at 50,000 × g for 20 min at 4°C. Then the supernatant was discarded, and the insoluble fraction was retreated twice in TNK buffer as described above to remove any nonspecific binding proteins. Final, the high-salt treated sample was suspended in 0.2 ml of TM buffer.

Low-pH treatment: Briefly, a 0.2 ml Triton-treated nucleocapsid suspension was centrifuged at 15,000 × g for 10 min at 4°C. The pellet was suspended in 0.5 ml of 0.1 M Glycine-HCl/pH 2.5 buffer by gently pipetting up and down. This process was repeated once to remove any remaining bound proteins. The low-pH treated sample was suspended in 0.2 ml of TM buffer.

### Expression and purification of proteins

VP51 and VP76 are the products of ORF *wsv*308 and *wsv*220 of WSSV (GeneBank accession no. AF332093) and composed of 466 and 674 amino acid residues, respectively. To prepare the specific antibodies against VP51 and VP76, a region of VP51 between amino acids 127 and 338 (212 amino acids, designated VP51p) and VP76 between amino acids 253 and 510 (258 amino acids, designated VP76p) were chosen for expression. The *vp51p *and *vp76p *were amplified from the genomic DNA of WSSV using the specific primers containing *BamH *I and *EcoR *I sites (underlined): 5'-GCAGGATCCAGTTTGTCCGGTGCGTAC-3'/5'-GCAGAATTCTGTTTCCTCAGCAGAACG-3' and 5'-GCAGGATCCGGCGATGATTCTGTAGATG-3'/5'-GCAGAATTCAGTACGTGCCCAACAAGC-3'. PCR products were digested with corresponding restriction endounclease and cloned into vector pET-His upstream of a 6-His tag (Gene Power Laboratory Ltd). The recombinant plasmids pET-VP51p and pET-VP76p were transformed into *Escherichia coli *strain BL21 (DE3) competent cells and confirmed by sequencing. For proteins expression, bacteria were cultured until the OD600 reached ~ 0.6, and induced with 0.4 mM isopropylthiogalactoside for 6 h at 37°C, then harvested by centrifugation.and. His-tagged recombinant proteins VP51p and VP76p were purified by using Ni-NTA metal-affinity chromatography under denaturing conditions according to the instructions of QIAexpressionist system (Qiagen).

### Antibody preparation and Western blot analysis

A polyclonal antiserum was prepared with purified recombinant protein by immunizing mice four times, each with an interval of 10 days. The antigen (~ 20 μg) was mixed and emulsified with an equal volume of Freund's complete adjuvant (Sigma), and then the emulsion was injected intradermally into mouse. Subsequent three injections were given using antigen emulsified with an equal volume of Freund's incomplete adjuvant (Sigma). Four days after the last injection, mice were exsanguinated, serum was collected and the titers of antibody were determined by enzyme-linked immunosorbent assay. Specific antiserum of high titer was stored in aliquots at -80°C until analyzed.

Protein samples from WSSV were subjected to SDS-PAGE in 12% gels and transblotted onto polyvinylidene fluoride membrane (Amersham Biosciences) by semi-dry blotting at a constant current of 0.5 mA cm^-2 ^for 1.5 h at room temperature. The membrane was immersed in blocking buffer (20 mM Tris-HCl/pH7.5, 150 mM NaCl, 3% BSA, 0.05% Tween-20) at room temperature for 1 h, followed by incubation with the specific antiserum (diluted 1:1000) in blocking buffer at 4°C overnight. Subsequently, a secondary antibody, alkaline phosphatase-conjugated goat anti-mouse IgG (Promega) was added at a dilution of 1:7500 in blocking buffer at 25°C for 1 h, and then signals were detected by a detection solution (50 mM Tris-HCl/pH 9.5, 100 mM NaCl, 5 mM MgCl_2_) containing NBT/BCIP (Roche).

### Immunoelectron microscopy (IEM)

WSSV virions or high-salt treated nucleocapsids were mounted on formvar-carbon-coated nickel grids grids (300-mesh) and incubated for 1 h at room temperature. After washing with PBS, the grids were blocked with 3% BSA in PBS for 1 h, followed by incubation with anti-VP51 or anti-VP76 serum (diluted 1: 200 in 3% BSA) for 2 h. After washing four times with PBS, grids were incubated with goat anti-mouse IgG conjugated to colloidal gold (10 nm; Sigma) for 1 h. Subsequently, grids were washed four times with PBS and briefly stained with 2% phosphotungstic acid (pH 7.0) for 20 min. Specimens were examined by TEM (JEOL 100 cxII). For control experiment, primary antibody was replaced with non-immune mouse serum and treated as above.

### Flow cytometry

A 0.2 ml WSSV virions or high-salt treated nucleocapsids were mixed with an equal volume of blocking buffer (50 mM Tris-HCl/pH7.5, 100 mM NaCl, 10 mM MgCl_2_, 3% BSA) and incubated for 30 min at room temperature, followed by incubation with anti-VP51 or anti-VP76 serum (diluted 1: 1000) for 1 h. Subsequently, the mixture was sedimented at 12,000 × g for 10 min. The pellets were washed thrice and resuspended in blocking buffer and incubated with fluorescein isothiocyanate (FITC)-conjugated goat anti-mouse IgG (diluted 1:1000) for 1 h, followed by centrifugation at 120,00 × g for 20 min, and then the pellets washed thrice with PBS and resuspension in 2 ml PBS. In order to validate the feasibility of the method, VP28 (known high-abundant envelope protein) or VP664 (known major nucleocapsid protein) was chosen as positive control. Anti-VP28 or anti-VP664 serum prepared in our laboratory (unpublished data) was used as primary antibody, whilst non-immune mouse serum was served as blank control. FITC-stained specimens were analyzed by flow cytometry (FACSCalibur^®^; Becton Dickinson) and fluorescence intensity was determined for each of the treatment groups. A total of 100,000 particles were analyzed in each experiment and the data are expression as the mean ± standard deviation of three independent experiments.

## Competing interests

The author(s) declare that they have no competing interests.

## Authors' contributions

CLW and FY conceived of the study, and participated in its design and coordination. They wrote the paper jointly.
